# Structural basis of the activation of MARTX cysteine protease domain from *Vibrio vulnificus*

**DOI:** 10.1371/journal.pone.0307512

**Published:** 2024-08-02

**Authors:** Lin Chen, Haider Khan, Lingchen Tan, Xiaojie Li, Gongchun Zhang, Young Jun Im

**Affiliations:** College of Pharmacy, Chonnam National University, Gwangju, Republic of Korea; Weizmann Institute of Science, ISRAEL

## Abstract

The multifunctional autoprocessing repeat-in-toxin (MARTX) toxin is the primary virulence factor of *Vibrio vulnificus* displaying cytotoxic and hemolytic properties. The cysteine protease domain (CPD) is responsible for activating the MARTX toxin by cleaving the toxin precursor and releasing the mature toxin fragments. To investigate the structural determinants for inositol hexakisphosphate (InsP_6_)-mediated activation of the CPD, we determined the crystal structures of unprocessed and β-flap truncated MARTX CPDs of *Vibrio vulnificus* strain MO6-24/O in complex with InsP_6_ at 1.3 and 2.2Å resolution, respectively. The CPD displays a conserved domain with a central seven-stranded β-sheet flanked by three α-helices. The scissile bond Leu3587-Ala3588 is bound in the catalytic site of the InsP_6_-loaded form of the Cys3727Ala mutant. InsP_6_ interacts with the conserved basic cleft and the β-flap inducing the active conformation of catalytic residues. The β-flap of the post-CPD is flexible in the InsP_6_-unbound state. The structure of the CPD Δβ-flap showed an inactive conformation of the catalytic residues due to the absence of interaction between the active site and the β-flap. This study confirms the InsP_6_-mediated activation of the MARTX CPDs in which InsP_6_-binding induces conformational changes of the catalytic residues and the β-flap that holds the N terminus of the CPD in the active site, facilitating hydrolysis of the scissile bond.

## 1. Introduction

*Vibrio vulnificus* is an opportunistic pathogen that can cause gastroenteritis, wound infection, and septicemia by consumption of contaminated seafood or exposure of skin wounds to contaminated water [1]. Three biotypes of *V*. *vulnificus* have been identified based on biochemical characteristics and phylogeny. Biotype 1 including MO6-24/O strain is responsible for the majority of ingestion cases and wound infections. Biotype 2 is a pathogen to farmed eels. Biotype 3 strains such as BAA87 cause wound infections and show hybrid characteristics of biotypes 1 and 2 [1, 2].

*V*. *vulnificus* secretes a large multifunctional-autoprocessing repeats-in-toxin (MARTX) as a main virulence factor via an atypical type I secretion system [3]. MARTX is a single polypeptide toxin consisting of central multiple effector domains and repeats-containing arms at the N- and C-termini [4]. The central region of the MARTX toxin of *V*. *vulnificus* MO6-24/O contains an autoprocessing cysteine protease domain (CPD) and four effector domains including the domain of unknown function (DUF1), the Rho-inactivating domain (RID), an αβ hydrolase (ABH), and makes caterpillars floppy-like domain (MCF) [4]. The MARTX toxins of the different *V*. *vulnificus* subtypes display a variation in the effector domains. CMCP6 and YJ016 strains have an additional Ras/Rap1-specific endopeptidase (RRSP) domain that dysregulates host cell signaling. The BAA87 strain does not contain MCF and RRSP but possesses ExoY-like adenylate cyclase domain (ExoY) and domain X (DmX) [4]. Upon binding to the host cell plasma membrane, the MARTX toxin forms a pore using repeat-containing arms and translocates the effector domains into the cytosol. The CPD directs the proteolytic processing of effector modules after activation by binding to the eukaryotic cell-specific inositol hexakisphosphate (InsP_6_) [5, 6]. The processing of the first cleavage site of the CPD exposes the active site to other cleavage sites within the toxin, subsequently generating active toxin fragments. DUF1 interacts with prohibitin 1 in the host membrane serving as an initial receptor for the binding of MARTX toxins. RID causes host cell rounding by inactivating Rho-family GTPases that regulate cell cytoskeleton. ABH is a phospholipase A1 intefering with the autophagic pathway and endosomal trafficking. MCF causes apoptotic cell death and Golgi disruption. Consequently, activated toxin effectors exhibit cytotoxic and hemolytic activities and induce host cell death [7–9].

The CPD displays a canonical caspase-like fold with a central β-sheet surrounded by α-helices. InsP_6_ binds to the CPD with the Kd of 1–2 μM and activates toxin autoprocessing [5]. InsP_6_ is abundant in the cytosol of mammalian cells at concentrations between 5–100 μM with a long half-life [10, 11]. InsP_6_ acts as an activator by binding to the site distinct from the active site of CPD. In addition, InsP_6_-binding stabilizes the CPD structure, facilitating the formation of the enzyme-substrate complex [6, 12]. The MARTX CPD of MO6-24/O strain shows a sequence similarity of 77% to the CPD of the BAA87 strain. The structure of MARTX CPD from *V*. *vulnificus* BAA87 biotype 3 was reported revealing attenuated activity of the CPD due to the distinct C-terminal β-flap region [13]. Currently, the MARTX CPD of the MO6-24/O strain is not known and all the reported structures of the MARTX CPDs have active conformations with InsP_6_-bound forms. The structure of apo-CPD with the inactive conformation is unknown.

Here, we present the high-resolution structures of the MARTX CPD–InsP_6_ complex of *V*. *vulnificus* MO6-24/O. Our results provide a structural basis for the InsP_6_-activated autoprocessing of CPD by rearrangement of the catalytic residues and the β-flap that holds the N-terminus of the CPD in the active site. After autoprocessing, the CPD adopts a postprocessing form that has a reduced affinity for InsP_6_ due to the loss of interaction between the β-flap and the N-terminal residues. The structure of the CPD Δβ-flap reveals an inactive conformation of the catalytic residues due to the absence of the interaction with the β-flap, providing a structural insight into the conformational switching of the CPD by InsP_6_-binding.

## 2. Materials and methods

### 2.1. Cloning of cysteine protease domain (CPD) of *V*. *vulnificus* MARTX

The DNA encoding the MARTX CPD (residues 3578–3796) was amplified by a polymerase chain reaction from the full clone of the MARTX toxin (NCBI code: WP_058645630.1) isolated from the MO6-24/O strain of *Vibrio vulnificus*. The MARTX CPD was subcloned into the BamHI/XhoI site of a modified pHIS2 vector. The MARTX CPD was tagged with the N-terminal hexahistidines followed by a thrombin protease cleavage site (LVPR/GS). The inactive mutant of the MARTX CPD was prepared by the point mutagenesis of the active site residue (Cys3727Ala). Other truncation constructs of the CPD were subcloned to the pHIS2 vector with the same procedure.

### 2.2. Protein expression and purification

*Escherichia coli* strain BL21(DE3) cells transformed with the plasmids encoding the MARTX CPD were grown to an OD_600_ of 0.8 at 37°C in LB medium. Cells were induced by the addition of isopropyl β-D-1-thiogalactopyranoside to a final concentration of 0.5 mM and were incubated for 12 h overnight at 20°C prior to harvesting. The cells expressing the CPD were resuspended in 2X PBS buffer containing 20 mM imidazole (lysis buffer) and lysed by sonication. The supernatant containing the His-tagged MARTX CPD was loaded to a Ni-NTA affinity column. The Ni-NTA column was thoroughly washed with the lysis buffer. The target protein was eluted from the column using a buffer containing 100 mM Tris-HCl pH 8.0, 300 mM imidazole, and 300 mM NaCl. The eluate was concentrated to 10 mg ml^-1^ using Amicon Ultra-15 centrifugal filter. The His-tag was cleaved by 10 international unit (IU) of thrombin protease (Reyon Pharmaceutical) per 10 mg of recombinant protein. The cleaved sample was subjected to size-exclusion chromatography (SEC) on a HiLoad Superdex 200 column equilibrated with 20 mM Tris-HCl pH 8.0 and 150 mM NaCl. The InsP_6_-loaded MARTX CPD complex for crystallization studies was prepared by supplementing 5 mM of InsP_6_ (*myo*-inositol hexakisphosphate, Merck) to the Ni-NTA affinity elutes and incubating for 1 h at room temperature before SEC. The fractions containing the MARTX CPD were concentrated by the centrifugal filter to 20 mg ml^-1^ for crystallization.

### 2.3. Crystallization and crystallographic analysis

For the crystallization of the InsP_6_-loaded MARTX CPD complex, the purified MARTX CPD (residues 3578–3796) was mixed with the additional five-time molar ratio of InsP_6_ and incubated at room temperature for 1 h. Preliminary crystallization experiments were carried out at 22°C in 96-well using customized crystallization screening solutions by dispensing 0.8 μl protein solution and 0.8 μl precipitant solution by hanging drop vapor diffusion method. The crystals of the pre-CPD-InsP_6_ complex were grown in 0.1 M HEPES pH 9.0, 30% PEG 8000 in 5 days.

The crystals of the post-CPD Δβ-flap were obtained using the construct (residue 3592–3796) lacking the N-terminal cleavage sequence. The C-terminal β-flap region of the post-CPD was susceptible to proteolytic degradation during protein purification. The purified post-CPD was mixed with the final 5 mM of InsP_6_ and incubated for 1 hour. The initial crystal appeared in a week in a solution consisting of 0.1 M Na-Acetate pH 4.6, 30% polyethylene glycol (PEG) 4000, and 0.1 M ammonium acetate. The crystallization condition was further optimized to 0.1 M sodium acetate pH 4.0, 25% PEG 3350, 0.2 M ammonium acetate, and 2.5% ethylene glycol via microseeding in 15-well screw-cap plates. A drop consisting of a 1.5 μl protein solution was mixed with a 1.5 μl precipitant solution and equilibrated against a 1 ml reservoir solution. The microcrystal seeds were introduced to the protein-precipitant mixture in an hour and the high-quality crystals with dimensions of 0.1 X 0.1 X 0.15 mm appeared in a week. The crystals of the MARTX CPDs were cryoprotected in a reservoir solution supplemented with 10% glycerol and flash-cooled by immersion in liquid nitrogen. Crystals were preserved in a cryogenic N_2_-gas stream (~100K) during diffraction experiments. Native diffraction data for the MARTX CPD were collected at a fixed wavelength of 0.97949 Å using an ADSC Q270 CCD detector on the 7A beamline at Pohang Light Source (PLS), Pohang Accelerator Laboratory. All data were processed and scaled using HKL-2000 [14]. The structure of the pre-CPD loaded with InsP_6_ was determined by molecular replacement using the structure of MARTX CPD (PDB code: 3EEB) as a search model. The single molecule of the MARTX CPD was found in the asymmetric unit using the program Phaser [15], and the density-modified map showed clear electron densities of the CPD and the bound InsP_6_. The final model was refined to R_work_ and R_free_ values of 17.6% and 19.3%, respectively using Phenix [16]. The structure of the post-CPD Δβ-flap was determined by molecular replacement using a truncated pre-CPD model. The crystal of the post-CPD did not contain the β-flap due to the proteolytic degradation during protein purification and crystallization. Two molecules of the CPD coordinating a single molecule of InsP_6_ in the dimer interface were found in the asymmetric unit. The models were built using the software Coot, and the final models were refined to R_work_ and R_free_ values of 20.7% and 27.7%, respectively (Table 1). The figures for all PDB structures were drawn using the software PyMOL (https://pymol.org).

**Table 1:** Data-collection and refinement statistics.

Crystal	MARTX pre-CPD–IP_6_ complex	MARTX post-CPD Δβ-flap
Construct	Residues 3578–3796, C3727A	Residues 3592–3796
Modeled residues	3583–3792	A chain: 3592–3762, B chain: 3590–3761
Data collection		
Beamline	PLS-7A	PLS-7A
Wavelength (Å)	0.97950	0.97950
Space group	*C2*	*P4*_*1*_ *2*_*1*_ *2*
Unit-cell parameters (Å, °)	*a* = 88.1, *b* = 69.8, *c* = 40.0 β = 107.7	*a* = 77.3, *b* = 77.3, *c* = 118.9
Resolution limit (Å)	50–1.3 (1.32–1.30)	50–2.2 (2.24–2.20)
No. of reflections	266543	219405
No. of unique reflections	54914 (2163)	18979 (918)
Multiplicity	4.9 (3.9)	11.6 (12.0)
Mean *I*/*σ*(*I*)	46.8 (7.5)	43.7 (7.6)
Completeness (%)	97.1 (76.4)	99.7 (99.9)
*R*_merge_ (%)	5.2 (26.3)	10.1 (54.1)
*R*_p.i.m._ (%)	2.3 (12.9)	0.030 (0.155)
Wilson *B* factor (Å^2^)	13.0	36.5
Refinement		
*R*_work_ (%)	17.6 (19.8)	20.7 (25.3)
*R*_free_ (%)	19.3 (20.9)	27.7 (34.2)
R.m.s.d., bond lengths (Å)	0.006	0.007
R.m.s.d., bond angles (°)	1.076	0.869
Overall *B* factor (Å^2^)	18.0	42.32
protein	16.5	42.10
ligand (InsP_6_)	13.4	58.83
water	25.7	41.99
No. of non-H atoms		
Protein	1647	2611
Ligand (InsP_6_)	36	36
Solvent	H_2_O 345, Na^+^ 1	H_2_O 102, Na^+^ 1
Ramachandran statistics		
Favored (%)	97.60	96.67
Disallowed (%)	0.48	0.00

### 2.4. In vitro autocleavage assay of the CPD

To monitor the InsP_6_-dependent autocleavage activities of CPDs, we used the N-terminal 6xHis-tagged CPD construct with a long N-terminal loop (residues 3570–3796) to observe the size difference clearly after cleavage by SDS-PAGE. The catalytically inactive mutant Cys3727Ala was used as a negative control. The 200 μl of 0.1 mM wildtype or mutant MARTX CPD was mixed with 0.5 mM InsP_6_ and incubated at room temperature with time intervals of 1 and 2 hours. To check the effect of InsP_6_ concentration on autoprocessing, 20 μM of the CPD was incubated with InsP_6_ concentrations ranging from 1 μM to 200 μM for one hour. The autocleavage was examined by SDS-PAGE.

### 2.5. Isothermal titration calorimetry

The InsP_6_-binding properties of the MARTX CPDs were quantitatively analyzed by ITC using an Affinity ITC calorimeter (low volume cell 190 μl; TA instruments). All proteins were prepared in the identical buffer containing 20 mM Tris-HCl pH 8.0 and 150 mM NaCl. The syringe was loaded with 1 mM of InsP_6_, and the cell was filled with 300 μl of 0.1 mM MARTX CPD. The titration curve was obtained by injecting 2 μl × 25 aliquots of the InsP_6_ into the cell at the time interval of 180 sec at 20°C. The enthalpy of the reaction, ΔH0, the binding constant, Kd, and the stoichiometry value, n, were calculated from the measured heat changes, δHi, upon the association of InsP_6_ and the MARTX CPD. The affinity Kd was estimated with a confidence level of 95% with a single-site binding model. The titration data were analyzed using the NanoAnalyze program (TA instruments) and fitted into an independent binding model.

## 3. Results

### 3.1. Structure of the pre-processed form of the MARTX CPD

**Fig 1:**
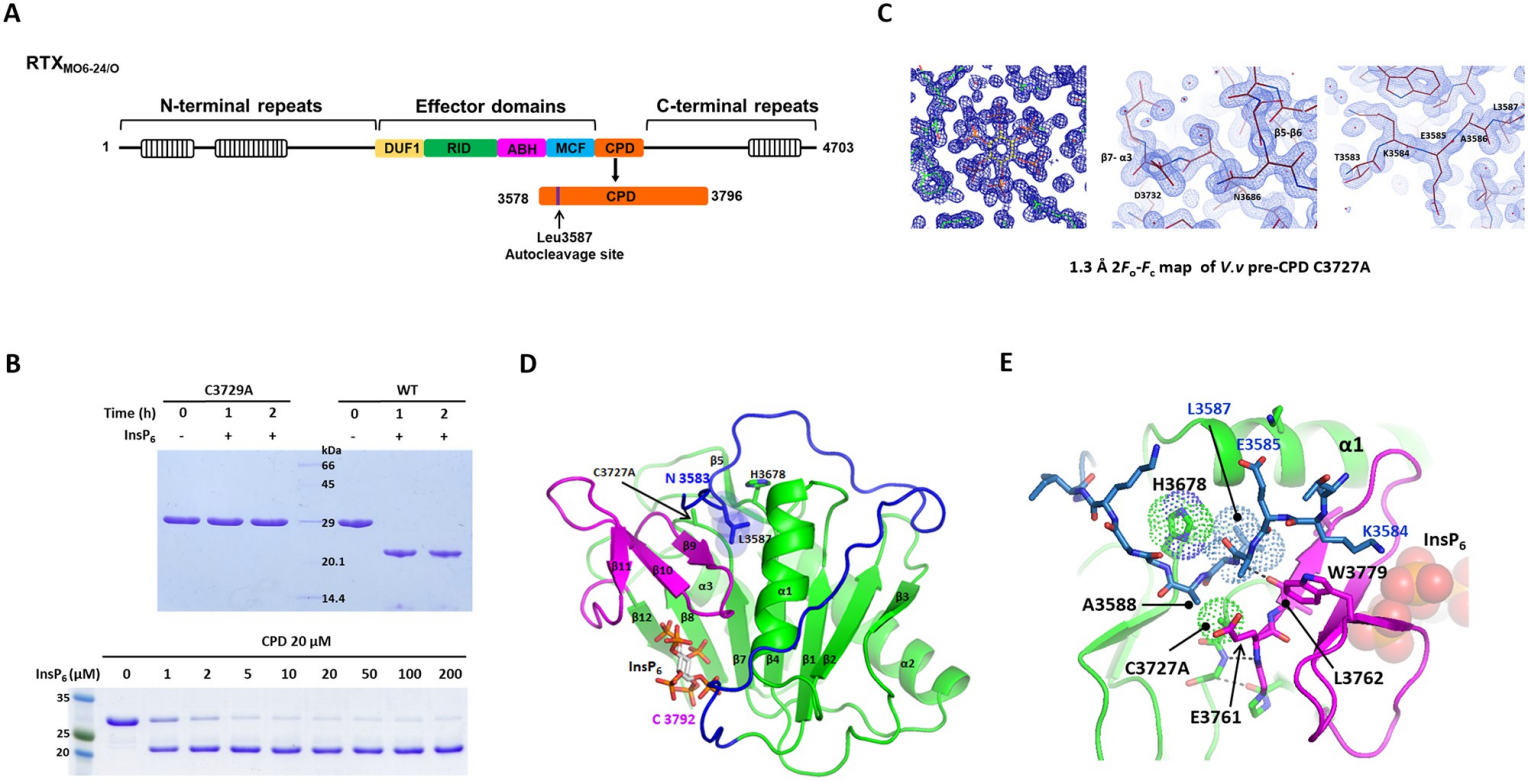
The overall structure of the *V*. *vulnificus* MARTX CPD. (A) Schematic representation of the domain structures of the MARTX toxin of *Vibrio vulnificus* strain MO6-24/O. The MARTX toxin contains a CPD (residue 3578–3796) and four effector domains including DUF1, RID, ABH, and MCF. (B) The purified CPD wild type (residue 3570–3796) shows autocleavage activity upon the addition of 0.5 mM InsP_6_. The active site mutant C3727A has no autocleavage activity. To examine the effect of InsP_6_ concentration on autocleavage, 20 μM of CPD was incubated with a series of InsP_6_ concentrations ranging from 1 μM to 200 μM for one hour. (C) The 2*F*o-*F*c electron density maps of the pre-CPD C3727A crystal with the final models superimposed. The three panels of density maps show the InsP_6_-binding site, N3686 of the β5-β6 loop, and the N-terminal five residues, respectively. (D) The overall structure of the pre-CPD C3727A bound with InsP_6_. The N-terminal leader, protease core, and β-flap are colored blue, green, and magenta, respectively, according to the annotation of Prochazkova *et al* [12]. The scissile residue, Leu3587 is shown as sticks with transparent spheres. (E) The N-terminal autocleavage sequence in the substrate-binding cleft. Leu3587 and the catalytic residues, His3678 and Cys3727Ala are shown with dotted sticks. Hydrogen bonds are shown in dotted lines.

*V*. *vulnificus* strain MO6-24/O contains a MARTX of 4703 amino acids with four effector domains including DUF1, RID, ABH, and MCF (Fig 1A). For the structural studies of the MARTX CPD, we purified the recombinant CPD (residues 3578–3796) containing the N-terminal ten residues upstream of the autocleavage site. The protein was expressed with an N-terminal His-tag and a thrombin protease recognition site for Ni^2+^-NTA affinity chromatography. The purified *V*. *vulnificus* MARTX CPD was a monomer in size exclusion chromatography (SEC). The recombinant CPD displayed InsP_6_-dependent autoprocessing activity. The incubation of wild-type CPD with 0.5 mM InsP_6_ resulted in autocleavage of the Leu3587-Ala3588 bond, while the active site mutant C3727A had no proteolytic activity (Fig 1B). The autocleavage was efficient in a low InsP_6_ concentration of 2 μM, when 20 μM of CPD was incubated for an hour at room temperature. To examine the activated conformation of the unprocessed CPD upon InsP_6_-binding, we crystallized the pre-processing form of CPD (here referred to as pre-CPD) bound to InsP_6_ using the catalytically inactive mutant C3727A. We determined the 1.3Å crystal structure of the CPD-InsP_6_ complex capturing the scissile bond harbored in the active site. The electron densities of InsP_6_ and the residues coordinating the ligand were well visible (Fig 1C). There was no residue in the disallowed region of the Ramachandran plot except Asn3686. The outlier residue, Asn3686 of the β5-β6 loop, showed well-defined electron density maps by interacting with Asp3732 of the β7-α3 loop (Fig 1C).

The *V*. *vulnificus* MARTX CPD displays a canonical CPD structure with the central seven-stranded β-sheet flanked by three α helices (Fig 1D). The core β-sheet of CPD is composed of 12 β-strands. The N-terminal five residues (TKEAL, 3583–3587) upstream of the scissile bond were ordered in the crystal structure (Fig 1C). The N-terminal leader of 34 residues (Thr3583-Arg3616) upstream of the β1-strand wraps around the CPD surface. The C-terminal β-flap composed of the three anti-parallel β-strands (β9-β11) is positioned between the N-terminal autocleavage sequence and the InsP_6_–binding site. The side chain of the scissile residue Leu3587 is inserted into the catalytic pocket formed between catalytic residues His3678 and Cys3727. The C_β_ atom of the mutated Cys3727Ala points toward the amide bond of the scissile bond, which represents the conformation ready for the catalytic reaction (Fig 1E).

**Fig 2:**
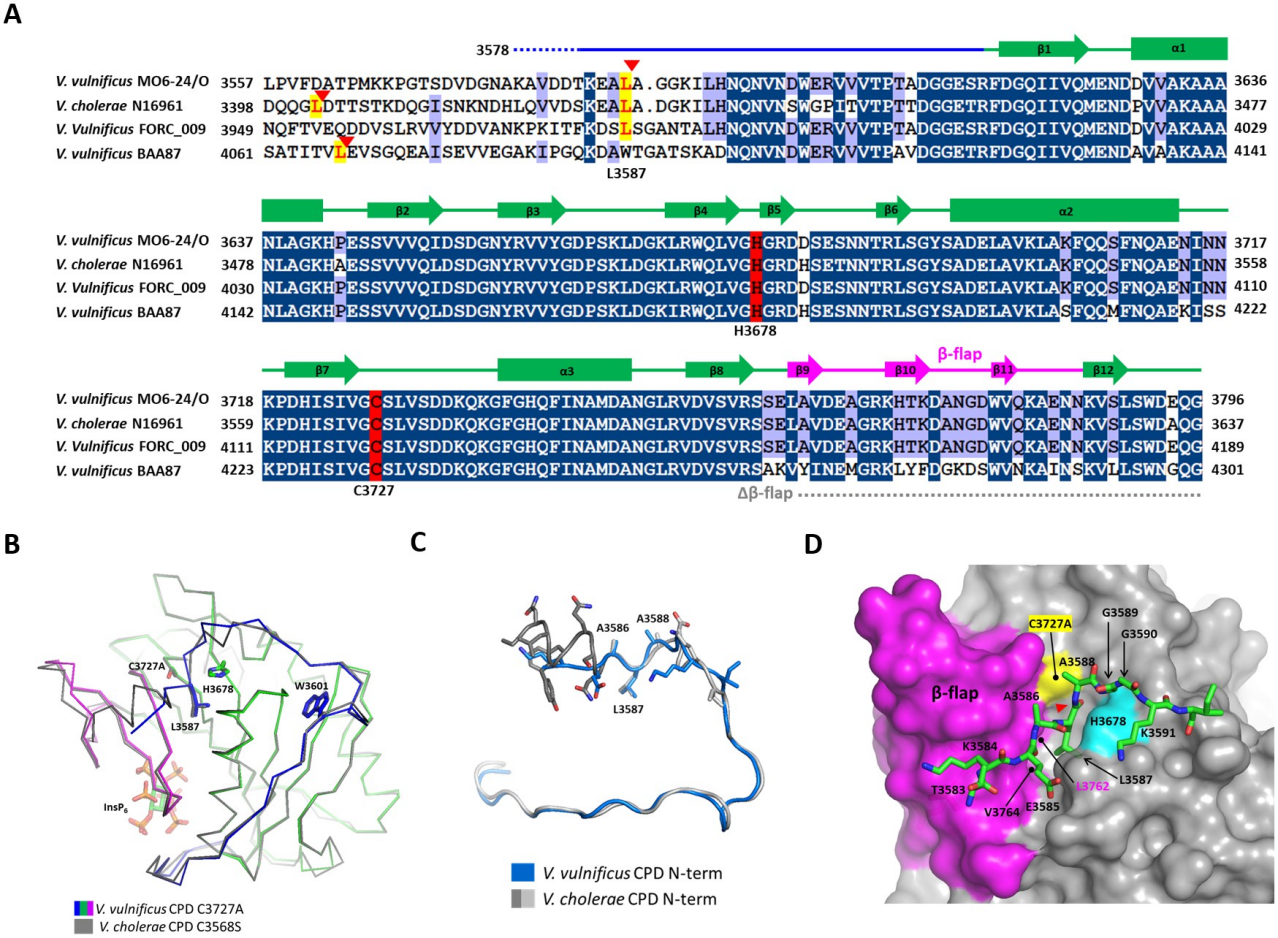
Structural comparison of the MARTX CPDs from different *Vibrio* strains. (A) Multiple sequence alignment of cysteine protease domains from MARTX toxins of various *Vibrio* strains including *V*. *vulnificus* MO6-24/O (WP_015728045.1), *V*. *cholerae* N16961 (AAD21057.1), *V*. *vulnificus* FORC_009 (WP_060534095.1), and *V*. *vulnificus* BAA87 (WP_039507922.1). The autocleavage sites in the N-termini of CPDs were indicated by yellow shades with red arrows. The dotted lines indicate the residues invisible in the electron density maps. (B) Structural comparison of *V*. *vulnificus* pre-CPD C3727A (this study) and *V*. *cholerae* pre-CPD (PDB code: 3FZY) [12]. The catalytic residues (Cys3727 and His3678) and the scissile residue (Leu3587) were shown for each CPD with stick models. (C) Structural comparison of the N-termini of *V*. *vulnificus* pre-CPD C3727A (this study) and the recombinant *V*. *cholerae* pre-CPD (PDB code: 3FZY). (D) The surface representation of the N-terminal autocleavage site. The N-terminal cleavage sequence is shown in green stick models. The active site residues, Cys3727Ala and His3678, are colored yellow and cyan, respectively.

The MARTX CPD of *V*. *vulnificus* MO6-24/O has sequence similarities of 87% and 77% to the CPDs of the *V*. *cholerae* N16961 and *V*. *vulnificus* BAA87, respectively (Fig 2A). The overall structure of *V*. *vulnificus* MARTX CPD is highly conserved to the structure of *V*. *cholerae* CPD with the C_α_ r.m.s.d. of 0.25 Å for 164 equivalent C_α_ atoms (Fig 2B). The scissile residues, Leu3587-Ala3588, and the active site residues of the *V*. *vulnificus* CPD have almost identical conformations compared to the residues of the *V*. *cholerae* CPD. The sequence upstream of the scissile bond is an extended loop in the *V*. *vulnificus* MARTX CPD. However, a TEV protease recognition sequence (ENLYFQS) in the corresponding region of the recombinant *V*. *cholerae* CPD forms an α-helix, suggesting that sequence variation in the N-terminal region can be accommodated around the substrate-binding cleft. (Fig 2C).

### 3.2. The binding of the N-terminal autoprocessing residues to the pocket

The crystal structure shows how the N-terminal substrate sequence is accommodated in the substrate binding cleft of the InsP_6_-bound CPD. The electron densities of the N-terminal four residues (3583–3586) upstream of Leu3587 were well visible (Fig 1C). The five residues of the autocleavage sequence at the P4, P3, P2, P1, and P1’ positions (P1 and P1’ refer to the N-terminal residue and C-terminal residue to the scissile bond, respectively), Lys3584 –Ala3588, are positioned between the helix ɑ1 and β9 of the β-flap in the pre-CPD. The scissile residue Leu3587 at P1 is inserted into the hydrophobic substrate-binding pocket (Fig 2D). The hydrophobic pocket is composed of five Val residues and one Leu residue. Val3764 and Leu3762 of β9 in the β-flap compose one side of the pocket wall. The backbone of Leu3762 in β9 makes a hydrogen bond with the amide nitrogen of the scissile residue Leu3587. The MARTX CPDs show a preference for small amino acids such as Ala or Ser in the P2 and P1’ positions to avoid steric clashes with Glu3761 of the β-flap (Fig 1E). The conserved residues at P4 and P3 positions also contribute to the substrate recognition in the pocket. Lys3584 and Glu3585 interact with the β-flap and the helix α1, respectively. The side chain stalk of Lys3584 at P4 makes hydrophobic interaction with Trp3779 of the β-flap. The side chain of Glu3585 at P3 interacts with the side chains of Lys3633 and Val3630 of α1.

The scissile bond is inserted between the catalytic dyad residues, His3678 and Ala-substituted Cys3727. The conformations of the catalytic and scissile-bond residues are almost identical to the structure observed for the *V*. *cholerae* MARTX CPD-InsP_6_ complex [5, 12]. The configuration of catalytic residues and the scissile bond suggests that the catalytic Cys is activated by the close alignment of the scissile bond followed by protonation of the leaving group by the catalytic His [17]. In conclusion, in the InsP_6_-bound form, the β-flap has a conformation that locks the N-terminus of the CPD in the active site by the interaction between the β-flap and the N-terminus, subsequently allowing hydrolysis of the Leu-Ala scissile bond.

### 3.3. The interaction of InsP_6_ with the β-flap activates the MARTX CPD for autoprocessing

**Fig 3:**
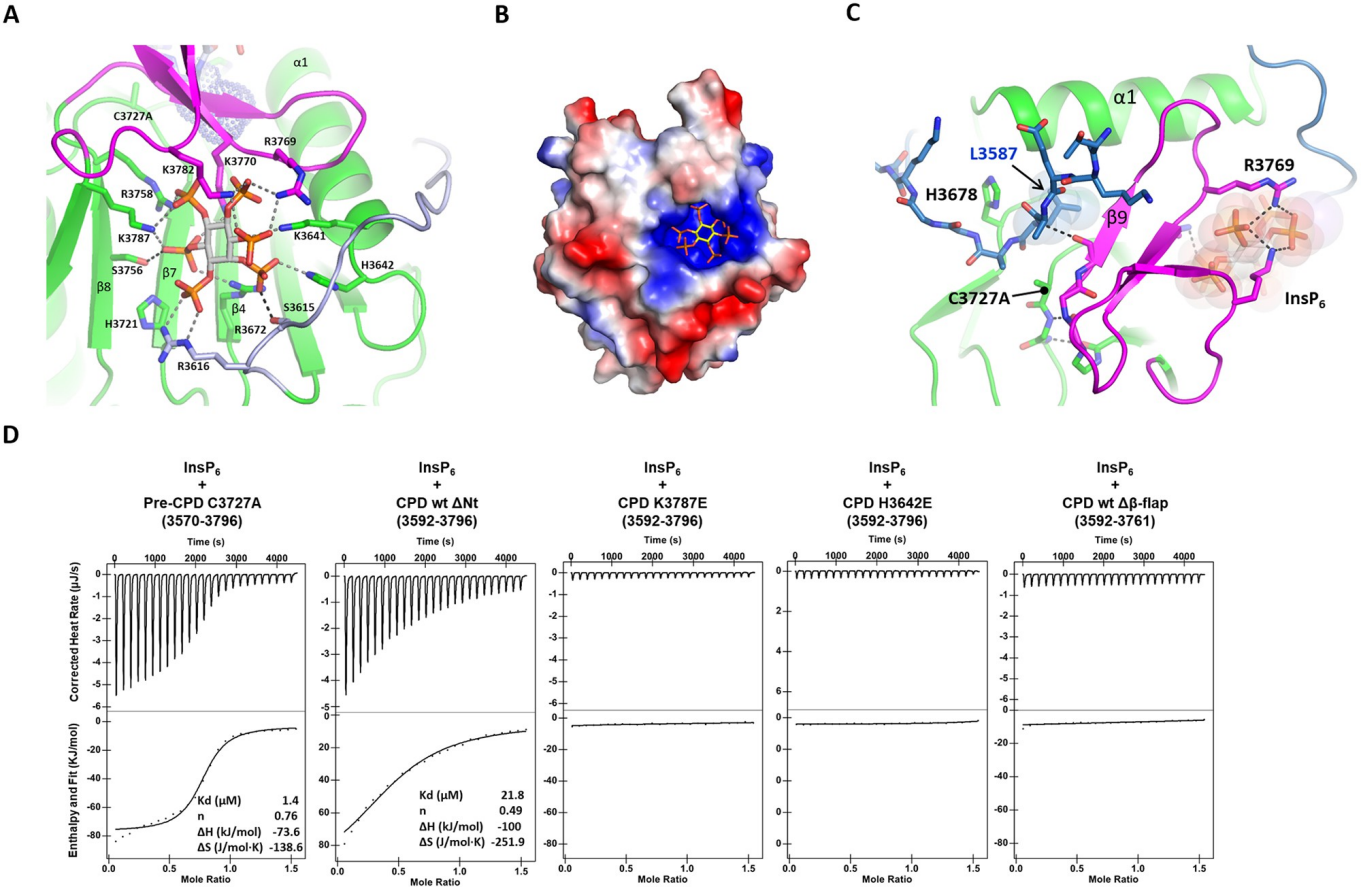
InsP_6_-binding to the basic pocket of the pre-CPD. (A) The InsP_6_-binding to the pre-CPD C3727A. The electrostatic interactions between InsP_6_ and the basic residues of the CPD are shown in dotted lines. The N-terminal cleavage sequence is colored in pale blue. The scissile residue Leu3587 is shown by dot representation. (B) Electrostatic surface representation of the basic InsP_6_-binding site of pre-CPD C3727A. The active conformation of the β-flap induced by InsP_6_-binding. The dotted lines indicate the hydrogen-bond networks of β-flap with the substrate, β7- α3 loop, and InsP_6_. (C) Measurement of binding affinities of InsP_6_ to the pre-CPD C3727A (3570–3796) and CPD ΔNt wt (3592–3796) by ITC. The CPDs of 0.1 mM were titrated with 1 mM of InsP_6_. The removal of the β-flap abolishes InsP_6_-binding and autocleavage activities.

InsP_6_ binds to the conserved basic pocket located distal to the substrate binding pocket. The three β-strands (β4, β7, and β8) of the core β-sheet form the floor of the binding pocket (Fig 3A). The β-flap and the N-terminal loop (residue 3612–3616) compose the wall of the binding pocket. InsP_6_ makes extensive electrostatic interactions with the binding pocket involving 331 Å^2^ of interacting surface area (Fig 3B). Totally 10 positively charged residues coordinate the phosphate groups of InsP_6_ (Fig 3A). InsP_6_-binding stabilizes the β-flap conformation required for the processing of the scissile bond. Three positively charged residues, Arg3769, Lys3770, and Lys3782 of the β-flap make ionic interactions with InsP_6_. The InsP_6_-binding to the basic pocket allosterically rearranges the conformation of active site residues by forming hydrogen-bonding networks of the β-flap with the active site and the substrate (Fig 3C). The β9β10 hairpin directly contacts both InsP_6_ and P1 Leu3587. The β9-strand of the β-flap forms one side of the pocket wall that accommodates Leu3587. The basic residues of the β10-strand interact with InsP_6_. The β8-β9 loop of the β-flap interacts with the β7-α3 loop locking the catalytic residue Cys3727 in an active conformation.

The binding affinity of InsP_6_ to the pre-CPD C3727A was 1.4 ± 0.5 μM measured by isothermal titration calorimetry (Fig 3D). The truncated CPD (residue 3592–3796) representing the post-processed form showed a reduced affinity to InsP_6_ with a Kd of 21.8 ± 5.0 μM. The data suggest that the pre-CPD containing the N-terminal cleavage sequence is configured for high affinity binding to InsP_6_. This is consistent with the reported InsP_6_-binding properties of the *V*. *cholerae* MARTX CPD. The pre-CPD of *V*. *cholerae* MARTX has an affinity to InsP_6_ with a Kd value of 0.6 μM [17]. However, the processed CPD of *V*. *cholerae* MARTX had a 500-fold reduced affinity to InsP_6_ and was reactivated for high affinity binding to InsP_6_ by cooperative binding of a new substrate [12, 13].

The mutation of the positively charged residues in the InsP_6_-binding pocket, K3787E or H3642E completely abolished InsP_6_ binding and autoprocessing activity (Fig 3D). The truncated construct lacking the β-flap (Δβ9-β12) was soluble, indicating that the β-flap is not essential for protein stability. However, the Δβ-flap construct completely lost InsP_6_-binding, indicating that the β-flap is essential for the InsP_6_-binding and catalytic activity (Fig 3D).

### 3.4. The CPD Δβ-flap displays an inactive conformation of the catalytic site

**Fig 4:**
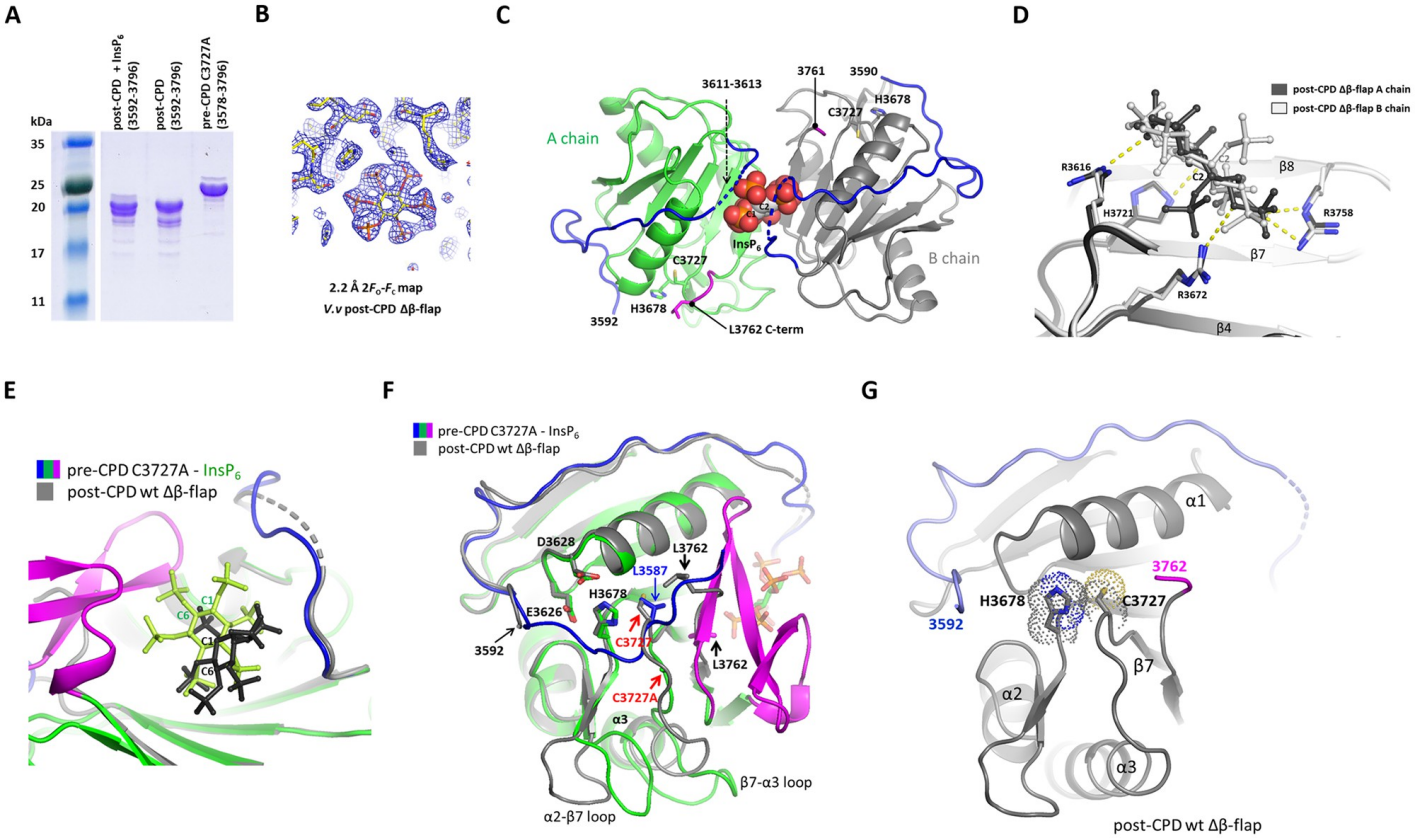
The structure of the CPD Δβ-flap in inactive conformation. (A) SDS-PAGE analysis of the crystallized CPD constructs. Lane 1, molecular weight marker; Lane 2, the purified CPD (residue 3592–3796) incubated with InsP_6_ was used for crystallization; Lane 3, the purified CPD protein (residue 3592–3796); Lane 4, purified pre-CPD C3727A (3578–3796). The purified proteins were incubated at room temperature for 48 h before SDS-PAGE analysis. The lane 1 and the other lanes were cut from the identical gel. (B) The 2*F*o-*F*c electron density maps of the InsP_6_ binding site in the post-CPD structure. (C) The structure of a dimeric CPD lacking the β-flap. InsP_6_ binds to the CPD in a 1:2 ratio. One of the protomers shown in gray is related by a noncrystallographic 2-fold axis. The disordered N-terminal loop (3611–3613) is shown in dotted lines. (D) Structural comparison of the InsP_6_-binding modes in the post-CPD Δβ-flap dimer. The identical InsP_6_ molecules bound to each promotor are overlayed to show the orientation of InsP_6_ to each protomer. The ionic interactions of InsP_6_ with the four basic residues were indicated by yellow dotted lines. (E) Structural comparison of the InsP_6_-binding to pre-CPD C3727A and the post-CPD wt Δβ-flap. (F) Structural comparison of pre-CPD C3727A and the proteolyzed CPD Δβ-flap. The equivalent catalytic residues, Cys3727, are indicated by red arrows. (G) Structure of the post-CPD Δβ-flap with the inactive conformation. The catalytic dyad residues, His3678 and Cys3727, are shown in dotted stick models.

After the autoprocessing of the scissile bond, the CPD is reported to adopt a conformation with reduced affinity to InsP_6_ [12]. To examine the structure of the processed CPD, we purified the wild-type CPD lacking the N-terminal autocleavage sequence (post-CPD, residue 3592–3796). The post-CPD construct in the absence of InsP_6_ was susceptible to proteolytic degradation of the C-terminal β-flap during protein expression and purification (Fig 4A). In contrast, the recombinant construct of the pre-CPD C3727A did not show significant proteolysis of the β-flap region. This observation indicates that the C-terminal β-flap region is flexible in an apo-form of the post-CPD due to the repulsion of positive residues in the InsP_6_-binding pocket. This is consistent with the previous report that the β-flap is resistant to limited proteolysis in the presence of InsP_6_ and the InsP_6_-binding induces stabilization of the β-flap in the activated CPD [6, 12].

Approximately 30 C-terminal residues were partially proteolyzed during protein purification, resulting in heterogeneity of the crystallized protein. The purified post-CPD construct was incubated with the final 5 mM of InsP_6_ for an hour before crystallization setup. InsP_6_-binding to the post-CPD was expected to stabilize the protein by forming electrostatic interactions between the β-flap and the core of the CPD domain. InsP_6_ displayed a binding affinity of 21.8 μM to post-CPD which is 16 times weaker than the affinity to pre-CPD. We obtained the crystals in a week at room temperature and determined the structure of the post-CPD at 2.2 Å resolution. The electron density maps were well-defined for the core of two CPDs and an InsP_6_ molecule (Fig 4B). However, the C-terminal β-flaps (residues 3762–2796) were not visible. In the asymmetric unit of crystals, there were two CPD molecules coordinating a single molecule of InsP_6_ in the center of the non-crystallographic two-fold axis (Fig 4C). The molecular packing in the crystal lattice does not allow the space for the folded β-flaps in the CPD dimer. Considering the proteolytic susceptibility of the β-flap, it seems that the crystals were formed by proteolyzed fragments lacking the β-flap (post-CPD Δβ-flap) or that the β-flaps were completely disordered. Two protomers have almost identical conformations except for the N-terminal disordered loops (residue 3611–3613). The dimer interface with 810 Å^2^ of a buried surface area is formed by the antiparallel interaction of β8 strands and α3 helices from each protomer. A single molecule of InsP_6_ in the crevice of the dimer interface interacts with the four basic residues of the InsP_6_-binding pockets from each protomer (Fig 4D). InsP_6_ does not bind tightly to the binding pocket due to the absence of the β-flap. Compared to the structure of the pre-CPD-InsP_6_ complex, the position of InsP_6_ in the post-CPD Δβ-flap was shifted 3 Å away from the binding pocket to share the coordination of a single InsP_6_ molecule by two protomers (Fig 4E). The recombinant construct (residue 3590–3762) of the CPD Δβ-flap corresponding to the crystallized fragment was monomer in SEC and had no InsP_6_-binding property, suggesting that the observed CPD dimer–InsP_6_ complex is a crystallographic artifact that was formed during the crystallization process.

Though the dimeric structure of the post-CPD Δβ-flap is physiologically not relevant, the monomeric structure seems to represent the inactive conformation of the core CPD when InsP_6_ is not loaded to the post-CPD. Compared to the pre-CPD-InsP_6_ complex, the post-CPD Δβ-flap shows large conformational differences in the α2-β7 and β7-α3 loops (Fig 4F). Due to the lack of interaction between the β-flap and the β7-α3 loop, the catalytic residue Cys3727 is displaced 5.4 Å toward the helix α1, occluding the substrate binding pocket (Figs 1E and 4G). However, the catalytic residue His3678 in the β4-β5 loop has no conformational difference. InsP_6_-binding contributes to enzyme activation by properly ordering the pocket and active site residues via interaction with the β-flap. Since the flexible β-flap directly contacts both InsP_6_ and the substrate, cooperative binding of InsP_6_ and a substrate to the post-CPD is required for catalytic activation.

In conclusion, InsP_6_-binding to the pre-CPD activates the cleavage of the N-terminal scissile bond, generating the post-CPD form. The post-CPD has a reduced affinity to InsP_6_ compared to the pre-CPD. When not loaded with InsP_6_, the post-CPD has an inactive conformation of catalytic residues and flexibility in the β-flap region due to the lack of interaction with a substrate and InsP_6_.

## 4. Discussion

*Vibrio vulnificus* can cause rapid and life-threatening sepsis and wound infections in humans. The virulence of *V*. *vulnificus* is governed by many factors including its acid resistance, capsular polysaccharide production, lipopolysaccharide, iron acquisition, cytotoxic factors, and expression of motility and adherence/adhesion molecules [18]. The MARTX toxin of *V*. *vulnificus* as a key virulence factor displays cytotoxicity and hemolytic activity [8, 9]. The inactivation of the *rtxA1* gene encoding MARTX significantly attenuates the virulence of *V*. *vulnificus* [9, 19]. Since the activation of the CPD is essential for the production of active MARTX effectors, the CPD was considered as a potential drug target for inhibition of MARTX activation. Small-molecule inhibitors against the CPD were developed to block the activation of *V*. *cholerae* MARTX toxin [12, 20]. However, the inhibition of CPD did not fully abolish the RTX activity and was not effective in blocking the action of the toxin in cells. Since the N terminus is bound within the active site prior to InsP_6_ binding in the pre-processed CPD, it occludes access of the catalytic Cys to protease inhibitors [12]. Upon autoprocessing of CPD, the exposure of the catalytic Cys to the CPD inhibitors facilitates the inhibition of subsequent processing of effector release. Alternatively, the activation of the CPD using InsP_6_ mimetics was considered a druggable approach by triggering the toxin auto-proteolysis prior to cell uptake and disrupting the translocation and activation process [6, 21]. Therefore, understanding the precise activation mechanism of the CPD might facilitate the development of new anti-toxin drugs targeting MARTX CPDs.

This study provides a structural basis for how InsP_6_-binding is communicated to the active site for activation of the *V*. *vulnificus* MARTX CPD by structure determination of the CPD–InsP_6_. The rearrangement of the β-flap by InsP_6_-binding locks the N terminus of the CPD in the active site and facilitates the hydrolysis of the Leu3587–Ala3588 amide bond. After autoprocessing, the CPD adopts a postprocessing form that has a reduced affinity for InsP_6_ due to the loss of interaction between the β-flap and the N-terminal substrate residues. The structure of the post-CPD Δβ-flap suggests that the substrate-binding pocket of the InsP_6_-free form is occluded due to the lack of interaction between the β-flap and the β7-α3 loop. The inactive structure of the post-CPD Δβ-flap correlates with the previous proposal that InsP_6_-binding regulates exposure of the active site [5, 6]. After the autoprocessing of the first scissile bond, the cooperative binding of both InsP_6_ and a new substrate to the post-CPD is required for catalytic activation by inducing a conformational change of the active site [12]. Then, the reactivated CPD cleaves the other sites between the effector domains of the MARTX toxin.

## Supporting information

S1 Raw image The original uncropped gel images of Figs 1B and 4B. (PDF)
