# Supplementary figures and images for: Structural basis of the activation of MARTX cysteine protease domain from Vibrio vulnificus
Source: PLoS One. 2024 Aug 2;19(8):e0307512. doi: 10.1371/journal.pone.0307512 (PMC11296635; doi:10.1371/journal.pone.0307512)

The original uncropped gel images of Fig 1B

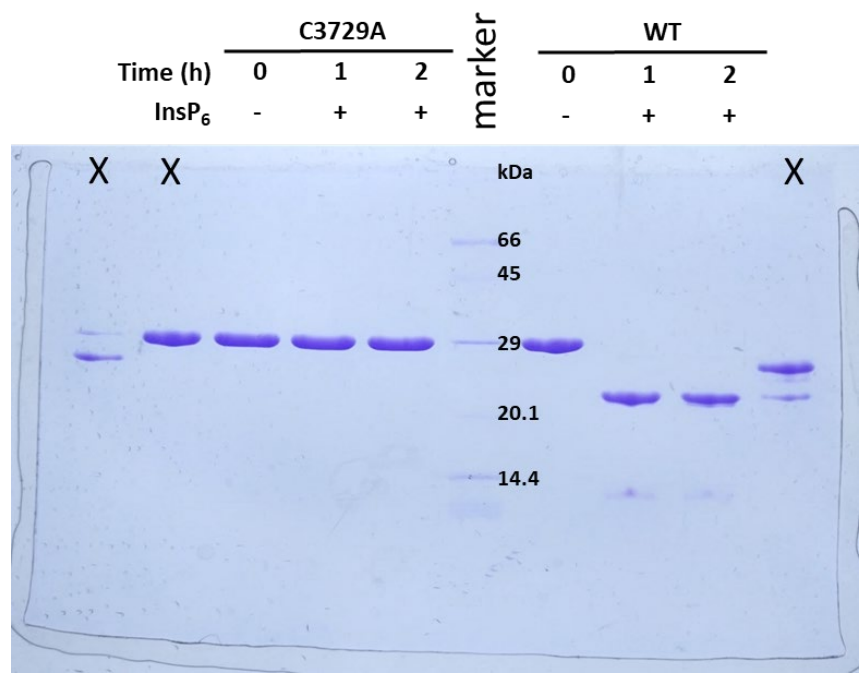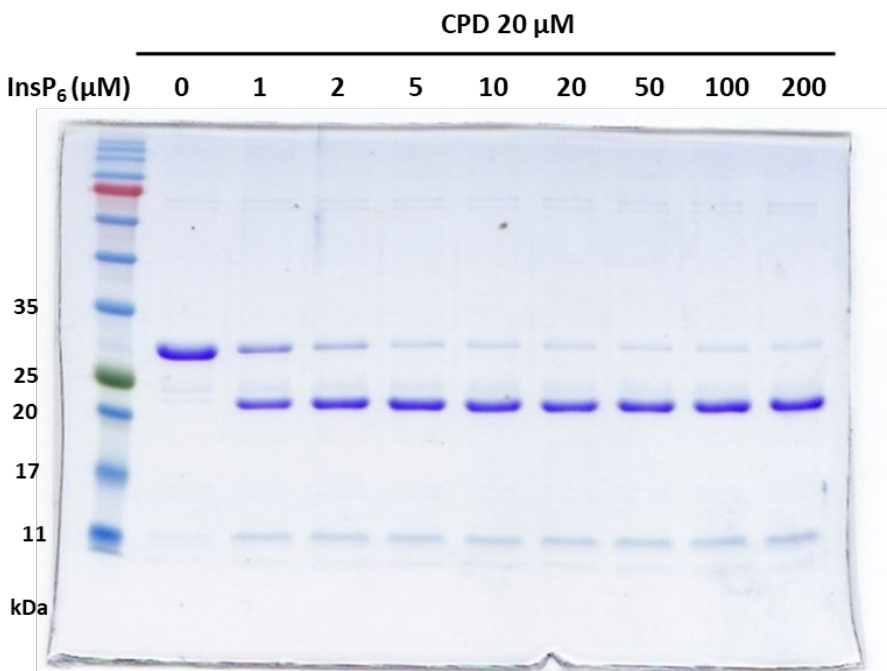

## The original uncropped gel image of Fig 4A

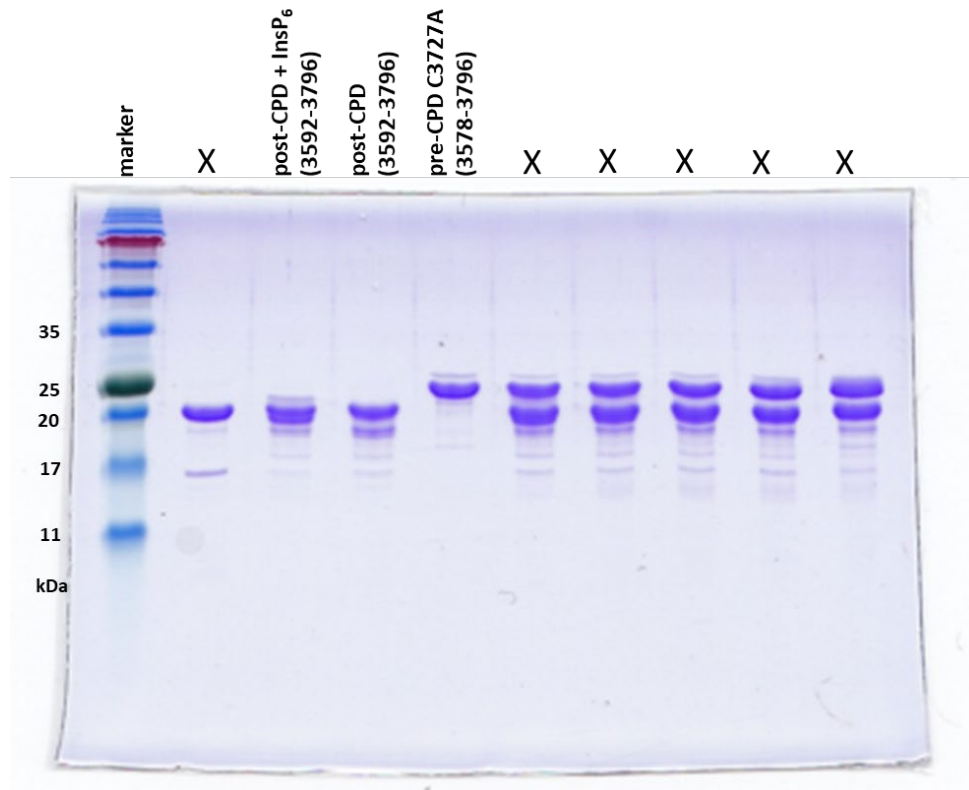

Supplement: S1 Raw image — (PDF) [file pone.0307512.s001.pdf]
